# Optimising Access to Healthcare for Patients Experiencing Homelessness in Hospital Emergency Departments

**DOI:** 10.3390/ijerph20032424

**Published:** 2023-01-30

**Authors:** Jane Currie, Amanda Stafford, Jennie Hutton, Lisa Wood

**Affiliations:** 1 School of Nursing, Queensland University of Technology, Kelvin Grove, Brisbane, QLD 4059, Australia; 2 Royal Perth Hospital, Perth, WA 6000, Australia; 3 Emergency Department, St Vincent’s Hospital Melbourne, Melbourne, VIC 3065, Australia; 4 Department of Medicine, Faculty of Medicine, Dentistry and Health Sciences, The University of Melbourne, Melbourne, VIC 3010, Australia; 5 Institute for Health Research, University of Notre Dame, Fremantle, WA 6061, Australia

**Keywords:** homelessness, access to healthcare, emergency department

## Abstract

The ED is often the first and sometimes the only place where people experiencing homelessness seek medical assistance. While access to primary healthcare is a preferable and more cost-effective alternative to ED, for many reasons, people experiencing homelessness are much less likely to have a regular General Practitioner compared to those living in stable accommodation. Drawing on a growing body of emergency care and homelessness literature and practice, we have synthesised four potential interventions to optimise access to care when people experiencing homelessness present to an ED. Although EDs are in no way responsible for resolving the complex health and social issues of their local homeless population, they are a common contact point and therefore present an opportunity to improve access to healthcare.

## 1. Introduction

In many developed countries, emergency departments are the frontline health providers for people experiencing homelessness. People experiencing homelessness have vastly higher mortality and morbidity rates compared with people who are housed [1] and a sobering three-decade gap in average life expectancy [2,3,4]. Disparities are also evident in patterns of healthcare access and use, with people experiencing homelessness being less likely to access preventive and primary healthcare services [5,6], which in turn contributes to higher rates of hospital use, including emergency departments.

Poor health is both a driver and a consequence of homelessness [7], and the enormous health inequalities experienced by people who are homeless are inextricably bound to the cumulative effect of social and structural disadvantages, collectively referred to as the social determinants of health [8,9]. These include poverty, financial insecurity, unsafe living conditions, insecure food availability, poor education and health literacy, unemployment, and the lack of social support [7]. This is very often layered with traumatic early life experiences [10], which are significant predictors of poor health outcomes and premature death [11]. Collectively, these factors fuel a vicious cycle of marginalisation and social disadvantage that are enormously deleterious to health [12]. In day-to-day survival mode on the streets, healthcare is often a low priority [5]. When people experiencing homelessness do access healthcare, they commonly experience stigma and discrimination [13], which further contribute to their ongoing disadvantage [10].

Upstream, reducing health inequalities associated with homelessness requires these and other social determinants to be addressed and prevented. More immediately, the most effective way to improve the health of people experiencing homelessness is, quite simply, to end their homelessness [12,14], with safe, stable, and affordable housing coupled with support services to keep them housed and by engaging them in regular, affordable healthcare in the primary care setting. Clearly, this is not within the remit of emergency department (ED) or hospital services and constitutes a problem for the whole of society and the whole of government. However, given this population’s high levels of hospital healthcare use, accessed via the ED [15], there is value in recognising that the ED is a major point of contact at which homelessness can be identified and interventions signposted or delivered.

## 2. Emergency Departments at the Frontline of Homeless Healthcare

The ED is often the first and sometimes the only place where people experiencing homelessness seek medical assistance [14,15,16]. Their ED presentations are often at later stages of an illness or injury or associated with acute complications of chronic diseases because they have not accessed earlier or preventive care due to practical, social, and financial barriers [17,18]. While access to primary healthcare is a preferable and a more cost-effective alternative to the ED, people experiencing homelessness are much less likely to have a regular General Practitioner compared to those living in stable accommodation [6,19].

Homelessness is strongly associated with a greater likelihood and frequency of ED visits, and this has significant capacity and cost implications for hospitals and healthcare systems [15,20,21,22]. Homelessness is also a consistent predictive factor for re-presentations to hospitals, a metric that is monitored as a healthcare performance indicator in many countries [21,22,23]. In one US study, for example, homeless patients were seven times more likely to return to the ED within 30 days [21], while in an Australian study, 43% of homeless patients reattended ED within 28 days [22]. ED presentations for this population are also more likely to lead to unplanned admissions, with a longer and hence more costly length of stay than the general population [22,24].

Frequent ED presentation and re-presentation by patients experiencing homelessness puts pressure on ED capacity and has an enormous price tag for the health system [21,24]. It is not surprising therefore, that people experiencing homelessness often present to ED with health issues that have escalated due to barriers to accessing far lower cost primary care and prevention [8,25]. Complex intersecting needs further complicate the ED presentations of people experiencing homelessness—these commonly include combinations of acute and chronic health issues, mental health, and substance use issues, along with significant social concerns which, obviously, include the lack of a safe place to stay.

Multimorbidity is ubiquitous in this population [26,27], making even simple medical issues difficult to manage. It is therefore unsurprising that a recent UK study reported that one-third of the premature deaths among people experiencing homelessness were attributed to health conditions that were preventable or treatable by standard medical care [2].

There is considerable tension between the complex needs of patients experiencing homelessness and the significant demands on busy EDs. From a patient perspective, a recent US study looking at ways to improve the care of homeless patients in the ED [28] reported that people often perceived that only their immediate medical issues were addressed, leaving them to be discharged back into homelessness without adequate consideration of wider underlying health conditions and social context [28]. As noted in a recent systematic review by Vohra et al. [15], the ED is an urgent care and resource-intensive environment, and it is challenging in this setting to care for patients with a multitude of diagnosed and undiagnosed health conditions, compounded by poor social circumstances. The COVID-19 pandemic has further added extraordinary pressure on strained hospital services, including EDs [29,30].

Nonetheless, despite ongoing and substantial demands on EDs and hospitals, it is rational and reasonable to reconsider the role of the ED in improving healthcare access for people experiencing homelessness. This is congruent with other emerging ED-demand-reduction initiatives that focus on identifying frequently presenting ED patient cohorts or those with specialist needs, such as mental health diversionary pathways [31] or geriatric assessment teams [32]. Indeed, a common issue seen in frequent ED presenters is multi-faceted problems that could often be more effectively addressed through long-term, community-based resources and supports rather than by EDs or hospitals. Indeed, it is often deficits in wider community-based systems or constraints on their capacity that drive recurrent ED presentations [12].

## 3. Recognising and Responding to Homelessness in the ED

Drawing on a growing body of emergency care and homelessness literature and practice, we have synthesised here some of the recognised and potential intervention touchpoints when people experiencing homelessness present to an ED. It is worth noting from the outset the potential applicability of many of these touchpoints across the broader hospital context.

### 3.1. Touchpoint 1: Waiting Environment

With few exceptions, hospital visits by people experiencing homelessness are via the ED, and planned or elective admissions are less common. Although EDs have the advantages of 24/7 access and free care at the point of delivery, they are a challenging environment for people experiencing homelessness. Given the high prevalence of trauma, substance use, anxiety, and other mental health conditions, it is not surprising that long waits, waiting amongst other people, or even the waiting area environment itself can contribute to agitation, anxiety, or premature departure from the ED. Therefore, people who are homeless are much more likely to leave the ED before being clinically assessed; one recent study found that almost one in five (18%) homeless patients left the ED prior to assessment [33]. Discharging themselves prior to completion of treatment is also more prevalent in this population than in others [34], and they often reattend later [22].

Stigma and judgment by others are often perceived by people with a lived experience of homelessness when they reflect on their ED experiences [35]. While there is often limited scope to alter the physical environment within the ED waiting area or triage process, attention given to providing a psychologically safe and trauma-informed environment can benefit many patients who are waiting to be seen, not only those who are homeless. Strategies to enhance psychological safety in the ED include ensuring privacy and safety from potential perpetrators in relation to domestic violence, and staff education to enhance confidence in providing trauma-informed care [36]. There may also be opportunities to support people experiencing homelessness while they are waiting to be assessed by a medical practitioner or nurse practitioner. This might include conducting some form of pre-screening assessment that identifies social support needs and then making early referrals to social workers and other specialist support services in the community, such as housing.

### 3.2. Touchpoint 2: Identifying Homelessness among Patients

International and Australian studies have shown that the prevalence of homelessness is under-recognised in hospital administrative data [15,22] and under-recognised by staff [28]. The identification of homelessness in ED is typically poor: for example, in a 2019 Australian study, specific screening for homelessness found that only 34% of instances of homelessness had been correctly identified in routine hospital administrative data [22]. Non-disclosure of homelessness due to fears of stigma or judgment (e.g., “trying to get a bed for the night”) is one potential contributing factor. Avoiding disclosure of homelessness by providing the address of emergency or shelter accommodation or a previous domicile is also common [15]. The way people are asked about homelessness is also important, however—neutral questions such as “do you have a safe place to sleep tonight?” can sufficiently identify homelessness and provides therefore an opportunity to offer referrals to appropriate support and housing services [37]. One option is that this information is gathered as part of the triage or administrative clerking so that it is routinely recognised at the start of the ED evaluation process. Ideally, a “social vulnerability” alert would be raised, either as an electronic flag or sticker on the ED notes. Another approach about to be trialled in several ED settings in Australia [19] combines screening for homelessness with an assessment of health-related vulnerability (burden of injury/illness combined with ability to access healthcare), with a decision assistance guide that informs clinicians decision making in case management, referrals, and on-ward care.

Screening for homelessness in EDs has been found to be acceptable for this population as long as privacy is respected [38]. For example, in a study that screened for the not unrelated issue of food insecurity, patients preferred to complete screening questions privately on a computer tablet over a verbal interview [39]. Lengthy screening tools can be burdensome in a busy ED context, and brief screening questions prevent disrupting the ED workflow but need to be evidence-based [40]. Recently a two-item self-rated questionnaire has been trialled and found to have acceptable sensitivity [41]. As screening for health risks or social factors may be perceived by patients as stigmatising, it is critical that any screening in an ED is performed in a non-judgmental way and avoids any risk of a negative experience for patients or staff [40]. It is imperative that there are existing systems to ensure that patients have adequate follow-up. Given these requirements, it is likely that a local screening system may need to be created that can service the ED population most appropriately. Improving the identification of homelessness in EDs, either by routine or targeted screening, is worthwhile for several reasons. Firstly, knowing a patient is homeless has important implications for emergency care, particularly for gauging the need for further medical assessments, suitability of treatment options, understanding the likelihood of treatment compliance, and making realistic and practical discharge plans [42]. Secondly, quantifying the true number of homeless patients in the ED is highly salient in establishing the frequency and impact of presentations on service provision, with a view to reducing the demand on EDs and ambulance wait times.

Thirdly, enumerating the prevalence of homelessness in EDs and hospital administrative data can bolster arguments for targeted specialist homelessness intervention, such as the Pathway model of a dedicated Homeless Team [43], which has been established in at least 14 public hospitals in the UK to date (pathway.org.uk). While the Homeless Team model varies between hospitals and is adapted to the local context, a key element is the hospital ‘in-reach’ of primary care and community-based homelessness expertise, working at the bedside with homeless patients in hospital, in the ED and in wards. Such teams comprise various combinations of staff, including Homelessness Medicine GP practices (GP, Nurse) and community homelessness organisations such as support and accommodation providers. This model has been adapted in Perth, Western Australia, with a dedicated Homeless Team with a GP, Nurse, and homelessness case worker in-reach on a daily basis to a large inner city hospital with a high (>5%) ED caseload of homeless patients (Royal Perth Hospital) [44]. Specialist homelessness services are also embedded in St. Vincent’s inner city public hospitals in Melbourne [45] and Sydney [46]. Beyond the demonstrated benefits of such hospital-based homelessness teams for patient care and reducing hospital utilisation [24,47,48,49,50], it also means that screening for homelessness feels less futile for ED staff because there is a specialist team to call when people experiencing homelessness are identified [16,24]. Having such a specialised service available in the hospital is of substantial benefit to work-pressured ED and other hospital staff and signals that homelessness is amenable to intervention [16].

### 3.3. Touchpoint 3: Linking People Experiencing Homelessness to Community Health, Housing, and Social Services

While a busy ED is unlikely to identify all health and psychosocial issues underlying the presentation of a homeless individual, there are opportunities to start this process in an ED visit or associated inpatient admission. Within the hospital, this could include referral to a social worker to address issues such as loss of ID, which can preclude access to accommodation or welfare benefits, or access to Family and Domestic Violence supports or refuge. Other examples of linkages that can be made within the health system include referrals to other healthcare providers for assessments or management of chronic issues, including medical and nursing or allied health specialists. It can also involve connecting people to important community services that may be able to organise and assess eligibility for sustained supports (such as disability benefits or housing support). These interventions can make a significant difference to this marginalised population, which often struggles with the bureaucratic processes required to receive the assistance needed to lift them out of their homelessness situation [42].

Identifying whether patients experiencing homelessness have a regular GP is important for continuity of care, as people experiencing homelessness are less likely to have a regular GP or to be engaged with primary care, yet paradoxically, have disproportionately high rates of chronic health conditions that are best prevented and managed outside of the hospital system [27]. However, there are few GPs with experience in Homelessness Medicine or with a willingness to take on homeless patients and provide no-cost consultations. This is an enormous barrier to the continuity of healthcare for this population group and pushes people experiencing homelessness towards ED presentations with the resultant problems for ED discharge planning and post-hospital follow up.

Last, but by no means least, is the importance of linkages to housing services, as long-term stable housing is quite literally the single most effective health intervention for people experiencing homelessness. Referring a patient to a housing service or Housing First program that can support them in accessing stable housing is a legitimate health intervention, as once housed, the likelihood of recurrent hospital use is reduced [44,51].

Critically, however, there has to actually be a sufficient supply of housing and accommodation available in the community, and this is a sticking point in ending homelessness in many cities and towns around the world. As noted recently by ED Physician Dr. Amanda Stafford, “governments are shooting themselves in the foot because by underspending on social basics like housing and support services, they end up overspending massively on more expensive healthcare” [12]. This speaks to the important advocacy role that hospital physicians have played in the literature and policy discourse as wider advocates for housing and support services, as well as the imperative to address socially determined drivers of both homelessness and health inequity [12,14,42].

### 3.4. Touchpoint 4: Discharge Planning

Discharge planning in the ED has an enormous impact on the effectiveness of the healthcare treatment provided in hospital, and on the likelihood of homeless patients returning to the ED in the near future [21,52]. Yet, the barriers posed by homelessness in relation to discharge are numerous. Where is a patient discharged to if they do not have a home? How are rest and recuperation possible? How can medications be safely stored or wound dressings kept clean? How can people be contacted for follow-up outpatient care or referrals without an address or a phone [53,54]?

Many pathways of care outside of EDs and hospital wards assume patients all have a supportive home environment where they can complete their treatment or recovery. Recognising the homeless status of patients in EDs thus allows for early referrals to social work and other supports within the hospital, as well as discharge planning, which incorporates links to homelessness and other support services [22,44,54]. Improved discharge planning for homeless patients reduces treatment failure, the likelihood of return visits to ED, and the need for subsequent hospital re-admission.

The ideal discharge option for hospitals with big local street sleeping populations, such as large inner-city hospitals, is a Medical Respite Centre (MRC); a supportive recovery environment can provide follow-up healthcare as well as social supports, including connecting people to housing or other accommodation. Having a medical respite option enables the safe and timely discharge of homeless patients, at a significantly cheaper cost per day than a hospital bed or repeated ED presentations [55,56]. Originating in the US, there are now over 120 respite centres for the homeless there, and now three in Australia, two affiliated with St Vincent’s Hospitals in Melbourne [55] and Sydney [57], and most recently, a 20-bed facility in Perth, Western Australia that has around the clock medical care on-site [58]. Evaluations of MRCs have shown substantial reductions in ED presentations and unplanned admissions by people experiencing homelessness [59]; hence a strong economic rationale, as well as a humane solution to avert discharges back to the street.

In the absence of an MRC, other pragmatic discharge interventions that hospitals can offer such as using brokerage money to provide short-stay accommodation post discharge or funding transport costs to return to family. The work of the Royal Perth Hospital Homeless Team has shown that this reduces the likelihood of recurrent hospital use [54]. The distribution of basic mobile phones to homeless patients in the ED allows them to be contacted for follow-up appointments and is a cheap way to improve outcomes [60].

## 4. Conclusions

The relationship between homelessness, health, and hospital use has often been described as a revolving door. Ultimately, the solutions to improving the health of people experiencing homelessness are anchored in social, economic, and structural factors beyond the remit of hospitals alone. Clearly, emergency departments are not responsible for resolving the complex health and social issues of their local homeless population, but the ED is the most common point of healthcare contact for many of the most vulnerable and marginalised people in our society. Given the frequency with which people experiencing homelessness present to EDs, we have presented here four ED touchpoints that hold the potential to improve healthcare access and outcomes for people experiencing homelessness. All are possible within the existing ED context, spanning the initial triage and waiting environment; the identification of homelessness among patients; linkages to other supports within the hospital and to housing, support and primary care services in the community; and considerations for discharge planning. Hospital collaborations with local community homelessness services and the introduction of evidence-based interventions such as the Pathway model of hospital homeless teams can further help to slow the revolving hospital door associated with homelessness.
